# Students Having Their Say: Novel Approaches and Solutions to Current and Emerging Public Health Problems

**DOI:** 10.5888/pcd22.250252

**Published:** 2025-08-21

**Authors:** Leonard Jack

**Affiliations:** 1 Preventing Chronic Disease, Office of the Director, National Center for Chronic Disease Prevention and Health Promotion, Centers for Disease Control and Prevention, Atlanta, Georgia

*Preventing Chronic Disease* (PCD) celebrates 14 years of providing students ranging from high schoolers to postdoctoral fellows with opportunities to develop their scientific writing and publication skills. Our Student Paper Contest (1) gives them the opportunity to serve as first and corresponding author on articles submitted to the journal for peer review. The contest has generated hundreds of submissions from around the world from students supported by their mentors to address an array of public health challenges. Student articles describe current and emerging public health challenges that include rural health, emergency preparedness, communicable disease, expansion of the capacity of future public health experts, strategies to prevent and reduce the impact of chronic disease, data modernization, and beyond.

PCD offers students the opportunity to submit articles for peer review in two ways. First, they can submit research papers for consideration in PCD’s Student Paper Contest, which welcomes submissions in two categories: original research and Geographic Information Systems (GIS) Snapshots. The contest has 5 primary goals (1):

- Provide students with an opportunity to become familiar with a journal’s manuscript submission requirements and the peer-review process
- Assist students in translating their knowledge and training in quality research into a journal’s publication requirements
- Develop students’ research and scientific writing skills to become producers, rather than just consumers, of knowledge
- Provide students with an opportunity to become a first author on a peer-reviewed article
- Promote supportive, respectful, and mutually beneficial student–mentor relationships that strengthen students’ ability to generate and submit subsequent scholarly manuscripts throughout their professional careers

As the Student Paper Contest evolved, PCD observed that not all students are interested in or have the capacity to conduct research, collect and analyze data, or produce quality research-related articles. Nevertheless, many are interested in generating articles that address the same important and timely public health issues in other formats. As a result, last year, we invited students to submit papers in a second category, the essay. Student submissions in this format are peer reviewed and must meet the following requirements:

1. Identify a public health challenge
2. Make the case for why it is a challenge
3. Discuss existing or historical approaches used to address the challenge
4. Identify innovative approaches and solutions
5. Describe what populations, groups, and/or organizations might benefit from the innovative approaches identified
6. Conclude by discussing what the future would look like as a result of uptake of the proposed innovative approaches

## 2025 Submissions

Nine student papers were accepted this year after undergoing careful peer review. This year’s collection consists of 8 essays and 1 original research article. No winners were selected with this year’s student paper contest. However, articles appearing in this year’s student paper collection address a range of timely topics:

- Use of smart-home technology to maintain and improve the mental health of older adults (2)
- A role for community pharmacies in treating tobacco and other nicotine dependence (3)
- Engaging young people in faith-based organizations to control hypertension (4)
- Promoting physical activity among college-age students (5)
- Increasing physical activity in schools (6)
- Addressing maternal illness and death in rural areas (7)
- The importance of dental care in preventing related chronic diseases (8)
- Human papillomavirus vaccination and screening in cervical cancer control (9)
- How adverse childhood experiences shape future health and cognitive decline (10)

## Conclusion

Over the years, PCD has taken proactive steps to create an environment that encourages students to develop their scientific writing skills. Hundreds of students have received extensive, thoughtful feedback, regardless of whether their papers were accepted for publication. Hence, we would like to congratulate every student who submitted a paper to the journal, this year and in all previous years, regardless of our acceptance decision. By submitting papers to PCD for consideration, students are positioned for future success through the intentional and consistent mentoring and extensive feedback they receive from faculty advisors, mentors, and professors, and the journal’s associate editors, editorial board members, and peer reviewers. We thank these volunteer experts for working alongside the journal to position all students for optimal growth and success.
